# Revealing short-term dynamics of tropical cyclone wind speeds from satellite synthetic aperture radar

**DOI:** 10.1038/s41598-024-61384-w

**Published:** 2024-06-04

**Authors:** Arthur Avenas, Bertrand Chapron, Alexis Mouche, Paul Platzer, Léo Vinour

**Affiliations:** Ifremer, Univ. Brest, CNRS, IRD, Laboratoire d’Océanographie Physique et Spatiale (LOPS), IUEM, 29280 Plouzané, France

**Keywords:** Natural hazards, Ocean sciences

## Abstract

Both unresolved physics in numerical models and limited theoretical understanding of the small-scale diffusion processes occurring near the ocean surface hamper predictability of tropical cyclone (TC) wind changes. An analytical model is here developed to diagnose the short-term evolution of the TC wind profile. An effective frictional parameter is introduced to control the unknown diffusion effects. When this frictional parameter is adjusted to match the TC intensity change, solutions are consistent with observed high-resolution ocean surface wind speeds from satellite synthetic aperture radar (SAR). The initial high-resolution estimate of the near-core wind structure is then found to strongly modulate the wind profile evolution. The frictional parameter can, unfortunately, not efficiently be calibrated using outer-core wind speed changes. Low-resolution observations or standard numerical weather predictions may thus not be directly used to reinterpret and anticipate short-term TC wind changes. The expected accumulation of orbiting SAR sensors as well as improved measurements of the ocean-atmosphere boundary layer characteristics shall then become essential to more precisely monitor TC dynamics.

## Introduction

Diagnosing short-term tropical cyclone (TC) wind profile changes is still very challenging. Numerical weather prediction currently faces limited capacity to address this difficult task^1–3^. Small-scale processes governing the TC dynamics may not be sufficiently well known and represented, especially when parameterized at coarse spatial resolution. Correcting biases in TC characteristics (intensity, radius of maximum wind) is thus an active field of research^4–7^.

However, TC dynamics may theoretically be described in a simple but comprehensive way, for both the steady^8–10^ and unsteady^11–14^ phase. Using high-resolution simulations^15,16^, and supported with observational data^17^, small-scale diffusion near the ocean surface is evidenced to alter the absolute angular momentum conservation and the TC wind structure. Analytical solutions for the steady TC phases can be adjusted to observed surface wind speeds to quantify small-scale turbulent exchanges^18^. The size of the TC core, controlled by small-scale diffusion, has been linked to unsteady phases^19,20^ and may support the diagnosis of the central pressure tendency^21^. Practical estimates of wind profile changes are then strongly constrained by the quality of observational data, especially near the TC core. Spaceborne scatterometers can be used, but surface wind speed estimates in the core region may often be limited by instrument resolution^22^, rain contamination and signal sensitivity issues^23,24^. TC core size estimates in best-track data is also debated, especially for the most intense TC systems^25^.

**Figure 1:**
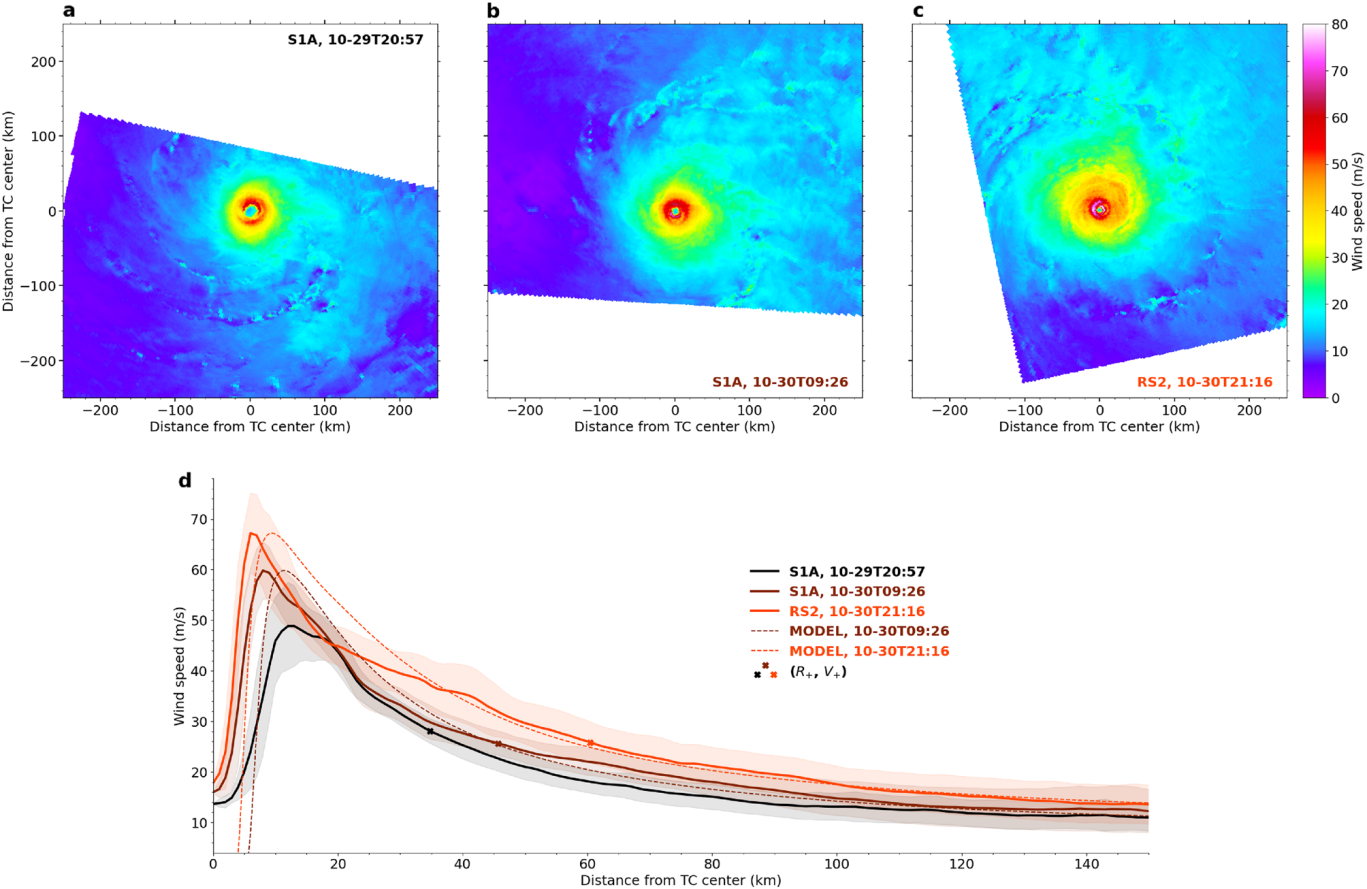
(**a–c**) SAR wind speed estimates for TC Goni in 2020 at three different times and (**d**) corresponding axisymmetric wind profiles (solid curves), standard deviation along each radius (shaded area), and analytical model predictions (dashed curves). For each wind profile, the cross mark indicates the radius $R_{+}$ of significant upward motions (see Eq. 2) and the corresponding wind speed $V_{+}$. Note, $h_{+} = 2.5$ km (see Eq. 9) for the first model prediction (brown dashed curve) and $h_{+} = 3.6$ km for the second model prediction (orange dashed curve).

In that context, satellite observation capabilities were extended by new acquisition modes and surface wind speed algorithms designed for spaceborne synthetic aperture radar (SAR) data. SAR observations of TCs now provide more accurate two-dimensional ocean surface wind speed estimates at very high-resolution ($\sim$1 km), including the inner-core region^24,26^. Figure 1a–c present three SAR acquisitions of TC Goni, a West Pacific system that reached category 5 on the Saffir-Simpson scale in 2020^27^. Successive acquisitions were taken at short time intervals ($\sim 12$ h), to examine the evolution of the TC axisymmetric wind profile (Fig. 1d) including the location ($R_{max}$) and amplitude ($V_{max}$) of its maximum.

Given these new observational opportunities, our motivation is to propose an analytical framework to help understanding and interpreting the short-term ($\sim 12$ h) evolution of the TC axisymmetric structure. Following a previous framework^14^, analytical solutions are extended for observed non-zero initial wind profiles to diagnose the TC evolution with a scalar parameter that characterizes the effects of frictional dissipation. After assessing the performance of the derived analytical solution compared to SAR data, its potential to enhance lower resolution tools is discussed. The benefit of future satellite capabilities to help estimating this frictional parameter is also emphasized, paving the way for future work on the monitoring and prediction of TC wind structural short-term changes.

## Theoretical framework

### Evolution of the wind profile

In the present work, the evolution of the TC wind profile is based on angular momentum conservation. When the radial circulation is prescribed (see for instance Eq. 9 from this study^14^), and considering a Rayleigh linear friction term, air parcels are governed by

1 $$\begin{aligned} \frac{\partial m}{\partial t} + u \left( \frac{\partial m}{\partial r} + fr \right) + \lambda m = 0, \end{aligned}$$

where $m = rv$ is the relative angular momentum, *r* the distance from TC center, *t* the time, *f* the Coriolis parameter, and *u*, *v* the radial and tangential components of the wind speed, respectively. The effective frictional parameter $\lambda$ has inverse time dimension and may be a function of *r*.

A natural characteristic time that normalizes Eq. 1 is $\frac{1}{f}$. Already using SAR observations and a theoretical framework, a previous study^28^ showed that a relevant characteristic length for TC dynamics is the radius $R_{+}$ of significant upward motions in the ocean-atmosphere boundary layer (BL), defined as

2 $$\begin{aligned} \omega _z(R_{+}) = 5 f, \end{aligned}$$

where $\omega _z(r) = \frac{1}{r} \frac{\partial m}{\partial r}$ is the relative vorticity. $R_{+}$ can be interpreted as the location where angular momentum is most efficiently gained by the TC system. Further normalizing the problem variables with the two characteristic scales *f* and $R_{+}$ (i.e *u* and *v* are both normalized by $f R_{+}$ and $\lambda$ by *f*), Eq. 1 is non-dimensionally reduced.

The radial wind is then imposed to take the following non-dimensional form

3 $$\begin{aligned} u = \left\{ \begin{array}{ll} -a r &{} \text {if } 0< r < 1 \\ -\frac{a}{r} &{} \text {if } r > 1 \\ \end{array}\right. , \end{aligned}$$

where *a* characterizes the inflow amplitude. With this definition, the absolute radial wind is maximal at $r = 1$, corresponding to $R_{+}$ in dimensional form.

Using Eq. 3 and a given initial arbitrary wind profile $v_0(r):= v(r, t = 0)$, Eq. 1 can be solved using the method of characteristics (see Text S1 in Supporting Information), yielding in non-dimensional form :

4 $$\begin{aligned} v(r, t)= \left\{ \begin{array}{lll} v_0(r e^{at}) e^{(a -\lambda ) t} + \frac{ar (1 - e^{(2a - \lambda ) t})}{\lambda - 2a} &{} \text {if } 0 \le r \le e^{-at} \\ \sqrt{1 + 2(\ln (r) + at)} v_0(\sqrt{1 + 2(\ln (r) + at)}) \frac{e^{-\lambda t}}{r} + \frac{a (\lambda r^2 - 2 a r^{\frac{\lambda }{a}})}{\lambda (\lambda - 2a) r} - \frac{a e^{- \lambda t}}{\lambda r} &{} \text {if } e^{-at} \le r \le 1\\ \sqrt{1 + \frac{2at}{r^2}} v_0(\sqrt{r^2 + 2at}) e^{-\lambda t} + \frac{a (1 - e^{-\lambda t})}{\lambda r} &{} \text {if }r \ge 1 \\ \end{array}\right. . \end{aligned}$$

Note, the linear effective friction term enables practical analytical solutions, Eq. 4, considered valid on a short enough duration, i.e for $t \sim \frac{1}{f}$.

### The effective frictional parameter

According to Eq. 4, the wind profile evolution solely depends on the initial distribution of winds $v_0$, the inflow amplitude *a*, and the effective frictional parameter $\lambda$. The latter shall describe the frictional influence of the BL on the flow. To further interpret this parameter, we recall the equation of angular momentum conservation in cylindrical coordinates for an axisymmetric vortex:

5 $$\begin{aligned} \frac{\partial m}{\partial t} + u \left( \frac{\partial m}{\partial r} + fr\right) + w \frac{\partial m}{\partial z} = \frac{r}{\rho } \frac{\partial \tau _{\theta z}}{\partial z}, \end{aligned}$$

where *z* is a vertical coordinate, *w* the vertical component of the wind speed, $\rho$ the density, and $\tau _{\theta z}$ a tangential stress component whose value at the ocean surface is assumed to be $C_d \rho v^2$, with $C_d$ a drag coefficient. In this cylindrical formulation, the frictional term $\frac{r}{\rho } \frac{\partial \tau _{\theta z}}{\partial z}$ varies with both *r* and *z*. In the present study’s framework, the frictional term $\lambda m$ affects an air parcel along its characteristic curve (see Text S1 in Supporting Information) and is thus expressed as a function of *r* only.

With the aim of reducing the frictional parameter $\lambda (r)$ to a scalar quantity, we propose to link its prescription in a framework based on the characteristic curves, used in the present work, with the cylindrical formulation (Eq. 5). We define the BL height *h* as the altitude where $\tau _{\theta z}$ vanishes. Averaging Eq. 5 over the BL depth and assuming a steady flow, we have^7^:

6 $$\begin{aligned} \overline{u} \left( \overline{\frac{\partial m}{\partial r}} + fr \right) = - \frac{C_d r v^2}{h}, \end{aligned}$$

where an overbar denotes a quantity averaged over the BL depth, *e.g* $\overline{u} = \frac{1}{h} \int _{0}^{h} u \, dz$. By analogy with this BL balance, the dimensional form of $\lambda$ may be assumed to satisfy:

7 $$\begin{aligned} \lambda m \propto \frac{\widetilde{C_d} r v^2}{h}, \end{aligned}$$

where the planetary part of angular momentum has been neglected for simplicity and $\widetilde{C_d}$ is an effective drag coefficient encoding the integrated effect of surface friction over the characteristic trajectory of air parcels. Note that the value of this effective drag coefficient $\widetilde{C_d}$ is expected to differ from the value of its cylindrical counterpart $C_d$ (Eq. 5).

Suggested by the potential vorticity conservation equation and aircraft wind speed measurements, it may be stated that the TC axisymmetric wind structure in the inflow is constrained by^7,29^

8 $$\begin{aligned} \widetilde{C_d} r v^2 = \text {cst}. \end{aligned}$$

Using Eq. 8 and further defining $h = h_{+} g(r)$, where $h_{+}$ is the value of *h* at $R_{+}$ and *g*(*r*) is a non-dimensional function of *r*, we may rewrite Eq. 7

9 $$\begin{aligned} \lambda \propto \frac{\widetilde{C_{d+}} R_{+} V_{+}^2}{h_{+} g(r) m}, \end{aligned}$$

where $\widetilde{C_{d+}}$ and $V_{+}$ are the effective drag coefficient and the wind speed both evaluated at $R_{+}$. In Eq. 9, the quantities $R_{+} V_{+}^2$ and *m* can be determined from the initial wind profile $v_0(r)$ so that, for a fixed function *g*(*r*) and corresponding value of $\widetilde{C_{d+}}$, determining $\lambda$ in Eq. 9 amounts to estimating a multiplicative constant which characterizes $h_{+}$.

## Data and methods

### Satellite data

The dataset of SAR high-resolution ocean surface wind speed estimates has already been described extensively^7,28^, and contains acquisitions from Sentinel-1A (S1A), Sentinel-1B (S1B) and Radarsat-2 (RS2) missions. Numerous studies demonstrated capabilities of spaceborne SAR C-band instruments to estimate ocean surface wind speeds under TC conditions, including at very high wind speeds and in the near-core region^24–26^.

Low-resolution ocean surface wind speed estimates from one passive L-band radiometer acquisition of the Soil Moisture Active Passive (SMAP) mission are also examined in the present work. The capacity of SMAP L-band brightness temperature measurements to retrieve ocean surface wind speeds in TCs has also been assessed in several studies^30,31^, and the consistency of such measurements with those from the SAR instrument evidenced^32^.

### Pairing the SAR observations

A pair of SAR acquisitions of the same TC system is retained only if the time difference between the two observations is within 10 and 14 h. To restrain the analysis to well-formed systems, we only select cases for which the SAR $V_{max}$ estimate (i.e the axisymmetric maximum wind speed) is higher than 25 m/s, the SAR $R_{max}$ estimate (i.e the axisymmetric radius of maximum wind) smaller than 150 km, and the absolute latitude of the TC center smaller than 30°. We also ensure that, for each SAR case, the distance to closest land from the TC center is greater than the SAR $R_{34}$ estimate (i.e the radius where the axisymmetric outer-wind profile equals 34 knots). Under these constraints, a dataset of 18 SAR pairs is created, with an average time difference between two successive acquisitions of 12.9 h.

### Adjustment of the analytical model

**Figure 2:**
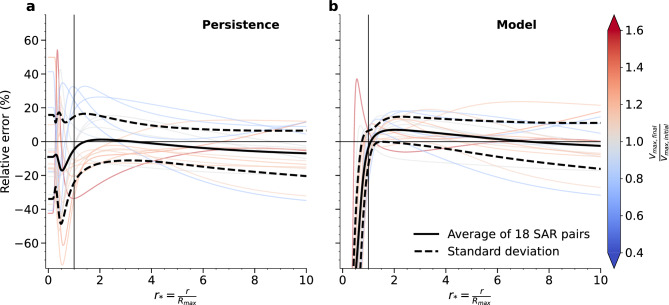
Relative error between (**a**) initial and final SAR wind profile estimates and (**b**) analytical model predictions and final SAR wind profile estimates of each SAR pair as a function of normalized radius ($r_{*} = \frac{r}{R_{max}}$ where $R_{max}$ is that of the final SAR wind profile). The different cases (thin curves) are colored by ratio of the final over the initial SAR $V_{max}$ estimates, while the average relative error (solid thick black curve) is displayed with plus or minus the standard deviation (dashed thick black curves).

For this study, *a*, *g*(*r*) and $\widetilde{C_{d+}}$ are chosen constant across all TCs in the proposed theoretical framework (Eqs. 3, 4, 9). When adjusting the analytical model, *a* is set to 0.5. Such a value was found to yield overall good performances of the analytical solution (see Fig. 2).

The definition of *g*(*r*) modulates the effective frictional parameter $\lambda$ in Eq. 9. Here, we impose a linear form $g(r) = r$. While determining a more appropriate definition for *g*(*r*) would improve the analytical solution, such an investigation should require a more extensive dataset of SAR acquisitions than what is available at the time of this study, and is thus left for future investigations. In addition, the simple linear definition still provides wind profile changes in the near-core region that are consistent with those observed in the SAR dataset of pairs (Fig. 2).

The value of $\widetilde{C_{d+}}$ must be consistent with realistic values of $h_{+}$ in Eq. 9. The height $h_{+}$ at which $\tau _{\theta z}(R_{+})$ vanishes can be estimated with the SAR dataset and ranges between $\sim$0.6 and $\sim$2.7 km, with a mean value of $\sim$1.4 km (see Text S2 in Supporting Information). When $\lambda$ is adjusted to the different SAR pairs to match the intensity change (see below), $\widetilde{C_{d+}}$ must be set to $1.2 \times 10^{-4}$ in order for the adjusted values of $h_{+}$ in Eq. 9 to be consistent with an average of $\sim$1.4 km.

The analytical model requires the estimation of $R_{+}$, which involves the computation of a radial derivative (see Eq. 2). Radial derivatives may be difficult to directly compute from SAR observations, *e.g* high wind speed estimates at high resolution may exhibit strong local variations (see for instance Fig. 1d). Hence, like in previous studies^7,28^, a parametric wind profile based on the Holland analytical model^33^ is adjusted to each SAR wind profile estimate. The adjusted parametric wind profiles are used to compute the quantities of interest (see below) as well as to perform comparisons (see Fig. 2).

For each pair of observations, the frictional parameter $\lambda$ is adjusted using the following procedure. The normalization constants *f* and $R_{+}$, as well as the quantities $V_{+}$ and *m* in Eq. 9 are all computed from the first (initial) acquisition of the SAR pair. The first SAR wind profile (parametric) estimate provides the initial condition $v_0$ in Eq. 4. Thereafter, the multiplicative constant $h_{+}$ that determines $\lambda$ is chosen so that, at the time of the second (final) acquisition of the SAR pair, the observed $V_{max}$ is matched by the analytical solution Eq. 4.

## Results

We assess the performance of the analytical solution (Eqs. 3, 4) when $\lambda$, prescribed by Eq. 9 and fully determined by the scalar quantity $h_{+}$, is adjusted to match the observed intensity changes. Considering the TC Goni observations, the two pairs cover $\sim 24$ h from its intensification phase. Figure 1d shows that TC Goni intensified from $V_{max} = 49$ m/s to $V_{max} = 60$ m/s and then to $V_{max} = 67$ m/s, while $R_{max}$ decreased from 13 to 8 km and then further to 6 km (solid curves). With the adjusted effective frictional parameter, the analytical model (dashed curves) is in qualitatively good agreement with the SAR wind profile estimates. The model predicts a $R_{max}$ value of 11 km (dashed brown curve) when taking the first (solid black curve) SAR wind profile estimate as initial condition, and 9 km (dashed orange curve) when the second (solid brown curve) SAR wind profile estimate is used as initial condition (Fig. 1d). While in good agreement with the SAR $R_{max}$ estimates, the small discrepancies in $R_{max}$ result in slight differences of wind speed estimates in that region. Note, the model wind speeds converge quickly toward zero with decreasing radius. This is a consequence of Eq. 9 and the linear assumption on *g*(*r*). To complement the analysis of TC Goni, four other case studies are presented in Text S3 in Supporting Information.

The analytical model is further assessed with respect to the complete dataset of SAR pairs, and compared to persistent expectations. Figure 2 presents the relative error for the persistence (i.e the prediction is just the initial SAR wind profile, Fig. 2a) and for the analytical model (Fig. 2b) as a function of normalized radius $r_{*}:= \frac{r}{R_{max}}$. To complement the analysis, the same comparison in terms of absolute error is presented in Text S4 in Supporting Information.

Note, for both the relative (Fig. 2) and absolute error (Text S4 in Supporting Information), the analytical model has a slight advantage over persistent conditions in that the maximum intensity is constrained by the observation.

The relative error considering persistent conditions (Fig. 2a) is low on average (black thick solid curve), especially for $1 \le r_{*} \le 5$, because the dataset consists in both weakening, stagnating and intensifying phases of TCs. When weakening phases (blue) are solely considered, the relative error is positive, as expected, and may be as large as $35\%$ in the region near $R_{max}$. Conversely, for intensifying phases (red), the relative error is negative, of the order $30\%$ near $R_{max}$. Lastly, the relative error is rather low for cases that have small $V_{max}$ variations (grey).

Regarding the analytical model (Fig. 2b), the average relative error is also low (black thick solid curve), but positive for $1 \le r_{*} \le 5$, suggesting that wind speeds are slightly overestimated by the model in this region. This is associated with a positive bias of the model in the prediction of $R_{max}$. In contrast to persistent predictions, there is no systematic bias specific to the phase of the TC life cycle (i.e weakening, stagnating or intensifying). Furthermore, the distribution of relative error values is narrower than that of persistent predictions (black thick solid curves). Near $R_{max}$ (i.e for $r_{*} \sim 1$), both the average relative error and the spread are small, suggesting that the analytical model performs better than persistence in this region.

Inside the core region (i.e for $r_{*} < 1$), the relative error takes large values for both predictions. Considering persistence, the large errors in this region are introduced by variations in $R_{max}$ between the initial and the final SAR wind profile estimates. For the analytical model, the relative error is largely negative, associated with the quick convergence of the analytical solution toward zero with decreasing radius. One case drastically deviates from this rule and has a relative error maximum of $37\%$ at $r_{*} \sim 0.5$. This case corresponds to TC Sam (see Text S3 in Supporting Information), for which the model overall captures the wind profile but fails to accurately reproduce the sharpness of the high winds region.

## Discussion

The systematic assessment of the model and the comparison with persistent conditions suggest that the adjusted analytical model captures the short-term evolution of the TC axisymmetric wind structure in a wide range of situations, especially near the TC core. In the present study, the effective frictional parameter is adjusted using both a high-resolution wind profile measured at the initial time step and an estimate of $V_{max}$ at the final time step. The question arises whether the frictional parameter could also be adjusted using information on the final outer-core wind profile, generally well captured by low-resolution measurements.

**Figure 3:**
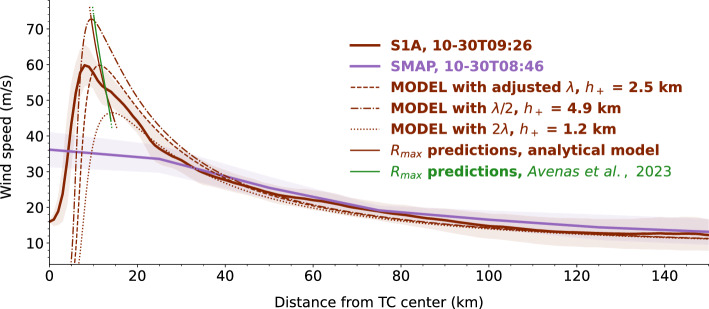
SAR wind profile estimate (solid thick brown curve, i.e the second of the three SAR wind profile estimates in Fig. 1) and radiometer wind profile estimate (solid thick purple curve) collocated in time (i.e with a 40-min time difference) for TC Goni. For each observed wind profile, the shaded area denotes the standard deviation along each radius. Analytical model predictions (thin dashed, dotted and dash-dotted brown curves) for three different values of $\lambda$ (see text for details) and corresponding $R_{max}$ estimates (thin solid brown curve, obtained using an ensemble of $\lambda$ values). For comparison, the $R_{max}$ estimates obtained by considering steady angular momentum conservation^7^ to the ensemble of analytical solutions (i.e the model $V_{max}$ and outer size predictions obtained when varying $\lambda$) are shown in green.

Figure 3 presents a wind profile estimate from a passive radiometer instrument (SMAP, purple solid thick curve) collocated with the SAR wind profile estimate from TC Goni (brown solid thick curve in Figs. 1d and 3). For radii larger than 30 km, both wind profile estimates are consistent. As expected near the TC core, the peak wind speeds are largely underestimated by the passive radiometer, mainly because of the coarse nominal spatial resolution ($\sim 50$ km) of the radiometer instrument^7^. Initialized on the previous SAR wind profile estimate (black solid curve in Fig. 1d, not shown in Fig. 3), the analytical solution is also displayed, once $\lambda$ was adjusted (i.e $h_{+} = 2.5$ km, brown dashed curve in Figs. 1d and 3), and when this value was doubled (i.e $h_{+} = 1.2$ km, brown dotted thin curve in Fig. 3) or halved (i.e $h_{+} = 4.9$ km, brown dash-dotted thin curve in Fig. 3). For these three values, the SAR and radiometer outer-core wind profiles are matched by the analytical model, but the corresponding $V_{max}$ estimates span a large range of values (between $\sim$47 and $\sim$72 m/s). For this case, the capabilities of current spaceborne passive radiometers or active scatterometers, which are limited when approaching the TC core, would not allow to adjust the frictional parameter. This suggests that information on the near-core surface winds is critical to diagnose the TC evolution.

Despite this wide range of predicted intensities, the range of model $R_{max}$ estimates (between $\sim$10 and $\sim$14 km, thin solid brown curve in Fig. 3) obtained when varying the effective frictional parameter is reasonably narrow and close to the actual SAR $R_{max}$ estimate ($\sim$8 km). Furthermore, the $R_{max}$ predictions from this ensemble of analytical solutions are consistent with those obtained by applying existing statistical rules^7^ (thin solid green curve in Fig. 3), also based on angular momentum conservation, to the ensemble $V_{max}$ and outer-core analytical estimates. Thus, for a given initial high-resolution wind profile, in the absence of any accurate $V_{max}$ estimate to calibrate $\lambda$, the analytical solution may still be used to create an ensemble of possible wind profile changes that shall be realistic in the outer-core region (see Fig. 2) and that are all physically consistent in the near-core region. More precisely, not only $R_{max}$ but also $R_{+}$ is well represented by the analytical solution (see Text S5 in Supporting Information). These two radii control the radial gradient of the near-core wind speed and thus modulate the TC steady-state balance^28^. Producing physically consistent estimates of $R_{max}$ and $R_{+}$ is thus essential to monitor the TC wind structure.

While the analysis suggests that the performances of the analytical model are reasonable, its limitations, such as the simple prescription of *u* (Eq. 3), the linear assumption on *g*(*r*), andthe use of angular momentum conservation to express the effective frictional parameter (Eqs. 5-9), should be kept in mind. These assumptions should certainly be revisited when more SAR data becomes available. Furthermore, deeper knowledge of the BL characteristics such as its actual height or the radial wind distribution would allow to further constrain the effective frictional parameter (Eq. 9) and the inflow amplitude *a*.

## Conclusion

An analytical solution for the short-term evolution of the TC axisymmetric wind structure, that relies on an effective frictional parameter, is developed and found consistent with observed high-resolution wind profiles. The frictional parameter is reduced to a scalar multiplicative constant and calibrated using an intensity change estimate. Seemingly, such a model adjustment could not efficiently be performed solely from the outer-core wind profile changes. The presented framework may then be used in at least two situations. First, to predict the complete wind profile at the current time, given a previous (*e.g* $\sim$12 h before) high-resolution wind profile estimate and a current intensity estimate (*e.g* from Dvorak analysis^34^). Second, to provide an ensemble of physically possible future wind profiles given a current high-resolution wind profile estimate and an ensemble of possible intensity change estimates^35^.

The proposed framework could also guide the analysis or reanalysis of the surface winds over a longer time period of the TC life cycle given a time series of $V_{max}$ estimates, by iterating the analytical model over several successive short time steps, starting from an initial observed high-resolution wind profile. Such intensity estimates could come from best-track reanalyses^36^ or objective analyses from spaceborne data^37^. Although the consistency between these intensity estimates and the SAR dataset may be high on average^25^, large discrepancies can occur for single cases. A consistent methodology to systematically calibrate the effective frictional parameter based on an ancillary intensity time series may thus still require further work.

The proposed simple analytical framework also informs on how future measurements of the surface winds and BL characteristics shall benefit the understanding of the TC wind structure evolution. In the coming years, satellite missions such as the Second Generation Meteorological Operational satellite program (Metop-SG), or the Harmony mission^38^ will provide improved TC ocean surface wind vectors estimates. Algorithms to estimate wind directions from the SAR sensors are also being developed, *e.g* based on local gradients analysis^39^. Airborne acquisitions from the Imaging Wind and Rain Airborne Profiler (IWRAP) instrument^40^ shall also yield useful information on both the BL depth and wind vectors. These BL measurements will then certainly help understanding how the small-scale processes modulate the frictional parameter (see Eq. 9).

Despite its simplicity, the proposed framework clearly emphasizes that reliable near-core surface wind speed estimates are crucial to anticipate changes in the TC wind structure. The expected accumulation of high-resolution observations due to the increasing number of spaceborne SAR sensors (*e.g* the recently launched Radarsat Constellation Mission) shall thus serve more in depth analysis of the TC dynamics^41^.

Lastly, the TC destructive potential is controlled by the complete wind structure^42^, while operational and research communities mainly focused on predicting intensity changes^43^. The proposed analytical framework may be practical in describing changes of the complete wind structure with only one scalar parameter, which efficiently characterizes the combination of an initial high-resolution wind profile and an intensity change. This shall in turn benefit the real-time evaluation of potential impacts (storm surges, waves, upwelling, currents) associated with an evolving TC.

### Supplementary Information

Supplementary Information.

## Data Availability

Datasets for this research are freely available online at https://cyclobs.ifremer.fr/app/tropical using the steps described at https://cyclobs.ifremer.fr/app/docs/.
