# Supplementary material for: Revealing short-term dynamics of tropical cyclone wind speeds from satellite synthetic aperture radar
Source: Sci Rep. 2024 Jun 4;14:12808. doi: 10.1038/s41598-024-61384-w (PMC11150253; doi:10.1038/s41598-024-61384-w)
Supplement: Supplementary file 1 — Supplementary Information. [file 41598_2024_61384_MOESM1_ESM.pdf]

Revealing short-term dynamics of tropical cyclone wind speeds from satellite synthetic aperture radar: Supporting information

Arthur Avenas[1,*], **Bertrand Chapron**[1], **Alexis Mouche**[1], **Paul Platzer**[1], **Léo Vinour**[1]

[1]Ifremer, Univ. Brest, CNRS, IRD, Laboratoire d'Océanographie Physique et Spatiale (LOPS), IUEM, F-29280, Plouzané, France
[*]arthur.avenas@ifremer.fr

Contents of this file

1. Texts S1 to S5
2. Figures S1 to S4

Text S1: Solution of the angular momentum equation

Here we solve the following angular momentum equation

$$\frac{\partial m}{\partial t} + u\left(\frac{\partial m}{\partial r} + fr\right) + \lambda m = 0 \tag{1}$$

for a given initial condition $m(r,t=0) := m_0(r) = rv_0(r)$ when the radial wind is prescribed by

$$u = \begin{cases} -ar & \text{if } 0 < r < 1 \\ -\frac{a}{r} & \text{if } r > 1 \end{cases} \tag{2}$$

Following the method of characteristics, we search a curve $(R(T),T)$ such that $R(t) = r$. Along such a curve, we have:

$$\begin{cases} \frac{dm(R(T),T)}{dT} = \frac{dR}{dT}\frac{\partial m(R(T),T)}{\partial r} + \frac{\partial m(R(T),T)}{\partial t} = -\lambda m - Ru \\ m(R(0),0) = m_0(R(0)) \end{cases} \tag{3}$$

where u takes the values given by Eq. 2 depending on the value of R. The piece-wise definition of u leads us to distinguish between three cases.

First case

We first consider a characteristic such that $0 \leq R(0) \leq 1$. With this condition, the characteristic satisfies the system

$$\begin{cases} \frac{dR}{dT} = -aR \\ R(t) = r \end{cases} \tag{4}$$

The characteristic is thus described by

$$R(T) = re^{a(t-T)} \tag{5}$$

The system satisfied by $m(R(T),T)$ becomes

$$\begin{cases} \frac{dm(R(T),T)}{dT} = -\lambda m(R(T),T) + ar^2 e^{2a(t-T)} \\ m(R(0),0) = m_0(re^{at}) \end{cases} \tag{6}$$

Solving this system leads to

$$m(R(T),T) = m_0(re^{at})e^{-\lambda T} + \frac{ar^2 e^{2a(t-T)}(1-e^{(2a-\lambda)T})}{\lambda - 2a} \tag{7}$$

Then, we evaluate this expression for $T = t$

$$m(r,t) = m_0(re^{at})e^{-\lambda t} + \frac{ar^2(1-e^{(2a-\lambda)t})}{\lambda - 2a} \tag{8}$$

Finally, we have

$$v(r,t) = v_0(re^{at})e^{(a-\lambda)t} + \frac{ar(1-e^{(2a-\lambda)t})}{\lambda - 2a} \tag{9}$$

This equation is valid when $0 \leq R(0) \leq 1$, or equivalently $0 \leq r \leq e^{-t}$.

Second case

We now consider a characteristic such that $R(0) \geq 1$ and $R(t) = r \geq 1$. With this condition, the characteristic satisfies the system

$$\begin{cases} \frac{dR}{dT} = -\frac{a}{R} \\ R(t) = r \end{cases} \tag{10}$$

The characteristic is described by

$$R(T)^2 = r^2 + 2a(t-T) \tag{11}$$

The system satisfied by $m(R(T),T)$ along such a characteristic then becomes

$$\begin{cases} \frac{dm(R(T),T)}{dT} = -\lambda m(R(T),T) + a \\ m(R(0),0) = m_0(\sqrt{r^2+2at}) \end{cases} \tag{12}$$

and is solved by

$$m(R(T),T) = m_0(\sqrt{r^2+2at})e^{-\lambda T} + \frac{a(1-e^{-\lambda T})}{\lambda} \tag{13}$$

Evaluating this expression at $T = t$ yields

$$m(r,t) = m_0(\sqrt{r^2+2at})e^{-\lambda t} + \frac{a(1-e^{-\lambda t})}{\lambda} \tag{14}$$

Finally, we write

$$v(r,t) = \sqrt{1+\frac{2at}{r^2}}\, v_0(\sqrt{r^2+2at})e^{-\lambda t} + \frac{a(1-e^{-\lambda t})}{\lambda r} \tag{15}$$

We recall that this equation is valid when $R(t) = r \geq 1$.

Third case

In the third case, a characteristic such that $R(0) \geq 1$ and $R(t) = r \leq 1$ is considered. Denoting T_1 the time when $R(T_1) = 1$, the characteristic satisfies the system

$$\begin{cases} \frac{dR}{dT} = -aR \\ R(t) = r \end{cases} \tag{16}$$

where $T \leq t$ is such that $R(T) \leq 1$. The characteristic is described by

$$R(T) = re^{a(t-T)} = e^{a(T_1-T)} \tag{17}$$

with

$$T_1 = \frac{ln(r)}{a} + t \tag{18}$$

The system satisfied by $m(R(T),T)$ is

$$\begin{cases} \frac{dm(R(T),T)}{dT} = -\lambda m(R(T),T) + ae^{2a(T_1-T)} \\ m(R(T_1),T_1) = m_0(\sqrt{1+2aT_1})e^{-\lambda T_1} + \frac{a(1-e^{-\lambda T_1})}{\lambda} \end{cases} \tag{19}$$

Solving this system yields

$$m(r,t) = m_0(\sqrt{1+2(ln(r)+at)})e^{-\lambda T} + \frac{a}{\lambda(\lambda-2a)}\left[\lambda r^2 e^{2a(t-T)} - 2ar^{\frac{\lambda}{a}} e^{\lambda(t-T)}\right] - \frac{ae^{-\lambda T}}{\lambda} \tag{20}$$

Taking $T = t$ in this expression, we obtain

$$m(r,t) = m_0(\sqrt{1+2(ln(r)+at)})e^{-\lambda t} + \frac{a(\lambda r^2 - 2ar^{\frac{\lambda}{a}})}{\lambda(\lambda-2a)} - \frac{ae^{-\lambda t}}{\lambda} \tag{21}$$

Finally, we have

$$v(r,t) = \sqrt{1+2(ln(r)+at)}\,v_0(\sqrt{1+2(ln(r)+at)})\frac{e^{-\lambda t}}{r} + \frac{a(\lambda r^2 - 2ar^{\frac{\lambda}{a}})}{\lambda(\lambda-2a)r} - \frac{ae^{-\lambda t}}{\lambda r} \tag{22}$$

This last equation is valid when $e^{-t} \leq R(t) = r \leq 1$.

Complete solution

Finally, the complete solution is

$$v(r,t) = \begin{cases} v_0(re^{at})e^{(a-\lambda)t} + \frac{ar(1-e^{(2a-\lambda)t})}{\lambda-2a} & \text{if} 0 \leq r \leq e^{-at} \\ \sqrt{1+2(\ln(r)+at)}\,v_0(\sqrt{1+2(\ln(r)+at)})\frac{e^{-\lambda t}}{r} + \frac{a(\lambda r^2-2ar^{\frac{\lambda}{a}})}{\lambda(\lambda-2a)r} - \frac{ae^{-\lambda t}}{\lambda r} & \text{if } e^{-at} \leq r \leq 1 \\ \sqrt{1+\frac{2at}{r^2}}\,v_0(\sqrt{r^2+2at})e^{-\lambda t} + \frac{a(1-e^{-\lambda t})}{\lambda r} & \text{if } r \geq 1 \end{cases} \tag{23}$$

Text S2: SAR h_+ estimates

Assuming $C_d r v^2 \approx$ cst, we can rewrite Eq. 6 from the main text

$$h_+ = -\frac{C_{d+} R_+ V_+^2}{\overline{u_+}\left(\left.\frac{\partial m}{\partial r}\right|_{R_+} + fR_+\right)}, \tag{24}$$

where C_{d+} and $\overline{u_+}$ are the drag coefficient and the averaged radial wind speed both evaluated at R_+. In Eq. 24, C_{d+} is set to 2.5×10^{-3} based on the literature[1], while f, R_+ and V_+ can be estimated from a SAR wind profile estimate. The radial gradient of relative angular momentum averaged over the BL $\left.\frac{\partial m}{\partial r}\right|_{R_+}$ is assumed to be close to that at the surface, so that it may also be computed on a SAR axisymmetric wind profile estimate. Because u_+ quickly decreases from the surface to the top of the BL, it can not be assumed independent from altitude in the BL. Instead, an exponential form may be assumed for the vertical distribution of the radial wind

$$u_+(z) = -\frac{V_+}{2} e^{-\frac{\alpha z}{h}}, \tag{25}$$

where α is constant and where we assumed that at the surface u_+ takes the value $\frac{V_+}{2}$. This value corresponds to a surface inflow angle of $\sim 26°$ at R_+, in agreement with *in-situ* measurements[2].
By taking

$$\alpha = \ln\left(\frac{V_+}{2afR_+}\right), \tag{26}$$

the expression also satisfies $u_+ = -afR_+$ at the top of the BL, which is an assumption of our model. Averaging Eq. 25 over the BL depth, we obtain

$$\overline{u_+} = \frac{V_+}{2\alpha}\left(e^{-\alpha} - 1\right). \tag{27}$$

Equations 24, 26, 27 can then be used to estimate h_+ for a given SAR wind profile estimate. Figure 1 displays the distribution of the SAR estimates of h_+ for the complete SAR dataset (grey bars) and for the SAR pairs considered in this study (blue bars). For the complete SAR dataset, the h_+ estimates range between $\sim$0.6 and $\sim$2.7 km, with a mean value of $\sim$1.4 km. For the dataset of 18 SAR pairs considered in this study, the h_+ estimates are higher on average, ranging between $\sim$1 and $\sim$2.7 km, with a mean value of $\sim$1.8 km. For the three SAR acquisitions of TC Goni, the h_+ estimates are $\sim$1.8, $\sim$1.4 and $\sim$1.3 km, close to the mean values from the two datasets.

Text S3: Application to four case studies

Four pairs of SAR wind profiles estimates and their corresponding predictions from the analytical model are displayed in Fig. 2. These case studies are described below.
Figure 2a presents a first case, TC Cebile, which evolved over the South-West Indian ocean in 2018 and reached category 4 on the Saffir-Simpson scale. During the beginning of its life cycle, TC Cebile was overflown by S1B on 2 January at 0022 UTC (black solid curve) and by S1A at 1323 UTC (orange solid curve). During this period, TC Cebile intensified from $V_{max} = 36$ m/s to $V_{max} = 45$ m/s, while the radius of significant upward motions contracted from $R_+ = 84$ km to $R_+ = 78$ km. For this case with a large and stagnating R_{max} $\sim$40 km, the analytical model (dashed orange curve) seemingly captures the wind speed increase in the near-core region, even though R_{max} is slightly overestimated, by $\sim$ 8 km. Within the inner-core region, *i.e* for radii below 30 km, the model underestimates the wind speed and quickly converges toward zero with decreasing radius.
A second case is presented in Fig. 2b and is that of TC Sam, a North Atlantic major hurricane of the 2021 season, which reached category 4 on the Saffir-Simpson scale. Two SAR acquisitions of TC Sam were successively performed by S1B and RS2 on 29 September at 2203 UTC and on 30 September at 0959 UTC, respectively. This period corresponds to a phase of rapid intensification, where V_{max} increased from 44 to 69 m/s, while R_+ contracted from 56 to 47 km. The increase of wind speed in the near-core region seems to be well captured by the analytical model. The stagnation of R_{max} at $23-24$ km is also caught by the analytical model, which predicts a value of 22 km. However, the sharpness of the high winds region is slightly

overestimated by the analytical model, so that, in the inner-core region, wind speeds are overestimated for radii between 10 and 20 km.
Fig. 2c displays a third case, TC Hector, which traversed the Pacific ocean during the 2018 season and reached category 4 on the Saffir-Simpson scale. During its life cycle, TC Hector was overflown by several spaceborne SAR instruments, including RS2 on 7 August at 1545 UTC and S1A on 8 August at 0414 UTC. This corresponds to a period when both V_{max} and R_+ were stagnating, at 52 m/s and 37 km, respectively. Accordingly, the two wind profiles estimated by the SAR instruments are similar. The analytical model well captures this stagnation phase in the near-core region, including the R_{max} which is located at $16-18$ km from the TC center.
A last case is showed in Fig. 2d and is that of TC Haleh, which evolved over the South-West Indian ocean in 2019 and reached category 4 on the Saffir-Simpson scale. During a weakening phase, TC Haleh was overflown by S1B at two successive times, on 6 March at 1354 UTC and on 7 March at 0049 UTC. During this period, V_{max} decreased from 41 to 30 m/s, while R_+ slightly increased from 70 to 73 km. The wind speed predicted by the analytical model is in agreement with the SAR wind profile estimate for radii larger than 60 km. However, the analytical model fails to capture the stagnation of R_{max} and predicts a value of 66 km, much larger than the $41-42$ km suggested by the SAR wind profiles estimates. As a consequence, the analytical model largely underestimates wind speeds for radii below 60 km. This last case illustrates how an error of the analytical model in the prediction of the radius of maximum winds might affect other parts of the wind profile.

Text S4: Absolute error of the analytical model

Figure 3 presents the absolute error for the persistence (Fig. 3a) and for the analytical model (Fig. 3b) as a function of normalized radius $r_* := \frac{r}{R_{max}}$.
Like for the relative error (see Fig. 2 from the main text), the absolute error considering persistent conditions (Fig. 3a) is low on average (black thick solid curve). When weakening phases (blue) are solely considered, the absolute error is positive and may be as large as 13 m/s in the region near R_{max}. Conversely, for intensifying phases (red), the absolute error is negative, of the order -20 m/s near R_{max}. Lastly, the absolute error is rather low for cases that have small V_{max} variations (grey).
Regarding the analytical model (Fig. 3b), the average absolute error is low (black thick solid curve) and positive for $1 \leq r_* \leq 5$. In contrast to persistent predictions, there is no systematic bias specific to the phase of the TC life cycle (*i.e* weakening, stagnating or intensifying). Furthermore, the distribution of absolute error values is narrower than that of persistent predictions (black thick solid curves). Near R_{max} (*i.e* for $r_* \sim 1$), both the average absolute error (2 m/s) and the spread ($\pm$2 m/s) are small.

Text S5: Model R_{max} and R_+ estimates

The performances of the analytical model in terms of radii R_{max} and R_+ are analyzed in Fig. 4. For R_{max} (Fig. 4a), the model estimates are in agreement with the final SAR estimates ($R^2 = 0.76$), although a small positive bias is apparent (~4.5 km). This bias results from the small positive bias of the model near the core region (see Fig. 3, or Fig. 2 from the main text). Remarkably, the performances of the model for R_{max} are much higher for the smaller R_{max} values, *i.e* the most intense cases.
Regarding R_+ (Fig. 4b), the model estimates are also in agreement with the final SAR estimates ($R^2 = 0.79$) and are negatively biased (~-4.0 km). This negative bias is small compared to the average R_+ value (~60 km).

References

1. Powell, M. D., Vickery, P. J. & Reinhold, T. A. Reduced drag coefficient for high wind speeds in tropical cyclones. *Nature* **422**, 279–283 (2003).
2. Zhang, J. A. & Uhlhorn, E. W. Hurricane sea surface inflow angle and an observation-based parametric model. *Mon. Weather. Rev.* **140**, 3587–3605 (2012).

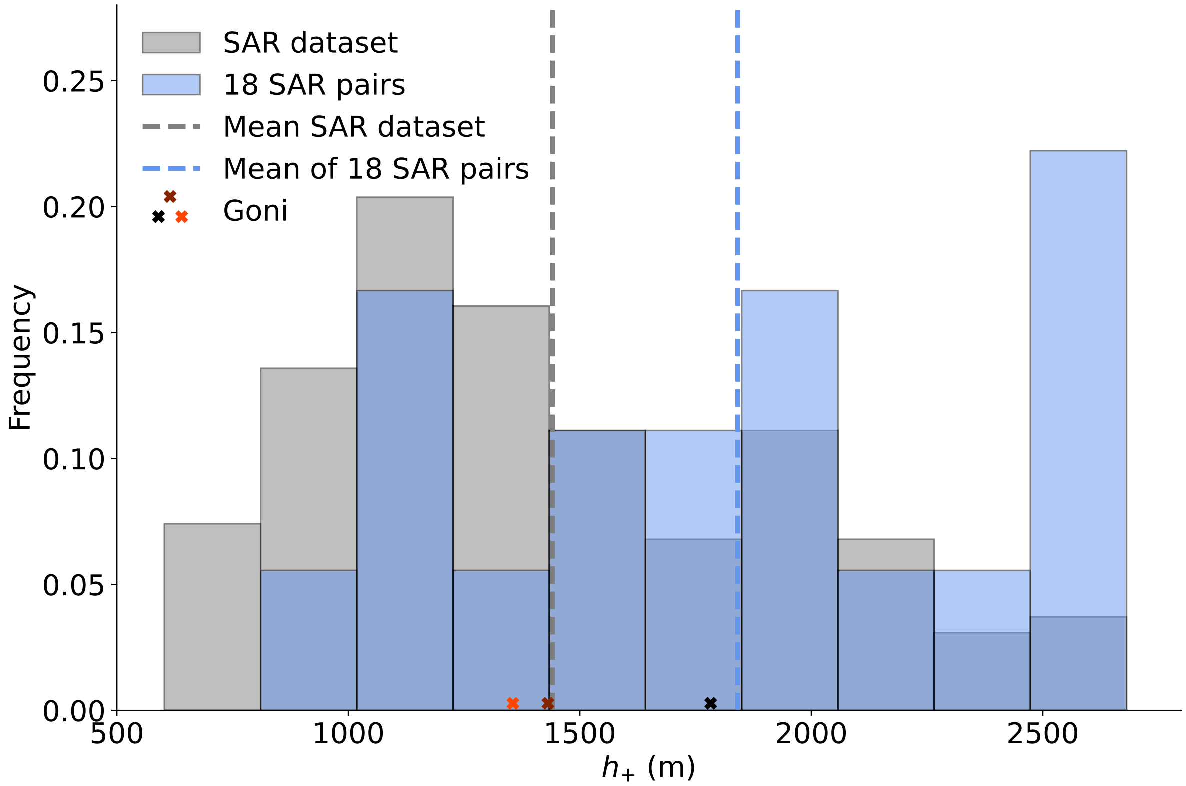

Figure 1. SAR estimates of h_+ after applying Eqs. 24, 26, 27 for the complete SAR dataset (grey bars) and for the dataset of 18 SAR pairs considered in the present study (blue bars). Dashed vertical lines correspond to the mean values from both datasets, while the three cross marks indicate the values corresponding to the three SAR acquisitions of TC Goni, following the color code used in Fig. 1 from the main text.

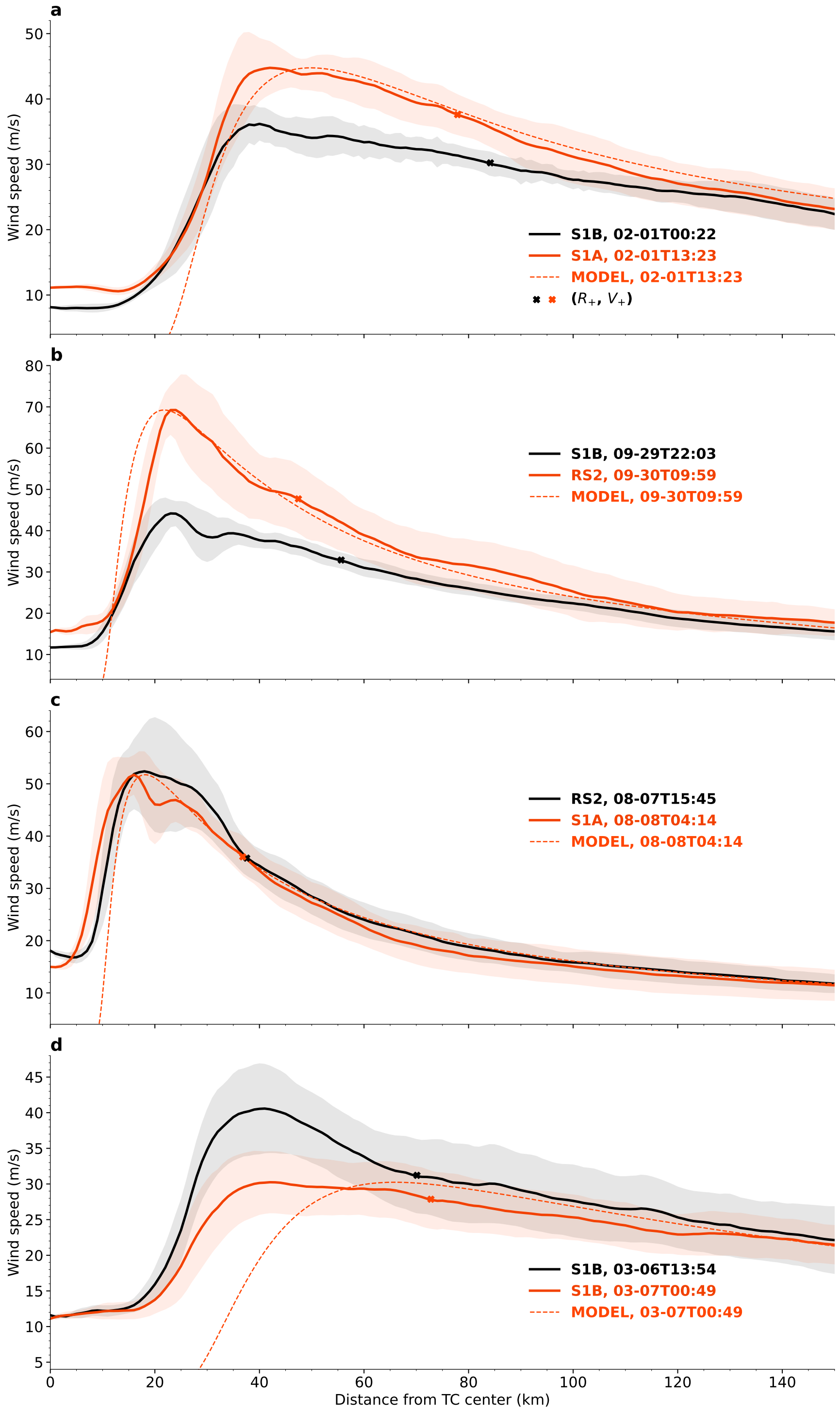

Figure 2. Initial (black solid curve) and final (orange solid curve) SAR wind profiles estimates, standard deviation along each azimuth (dashed area), as well as analytical model predictions (orange dashed curve) for (a) TC Cebile in 2018, (b) TC Sam in 2021, (c) TC Hector in 2018 and (d) TC Haleh in 2019.

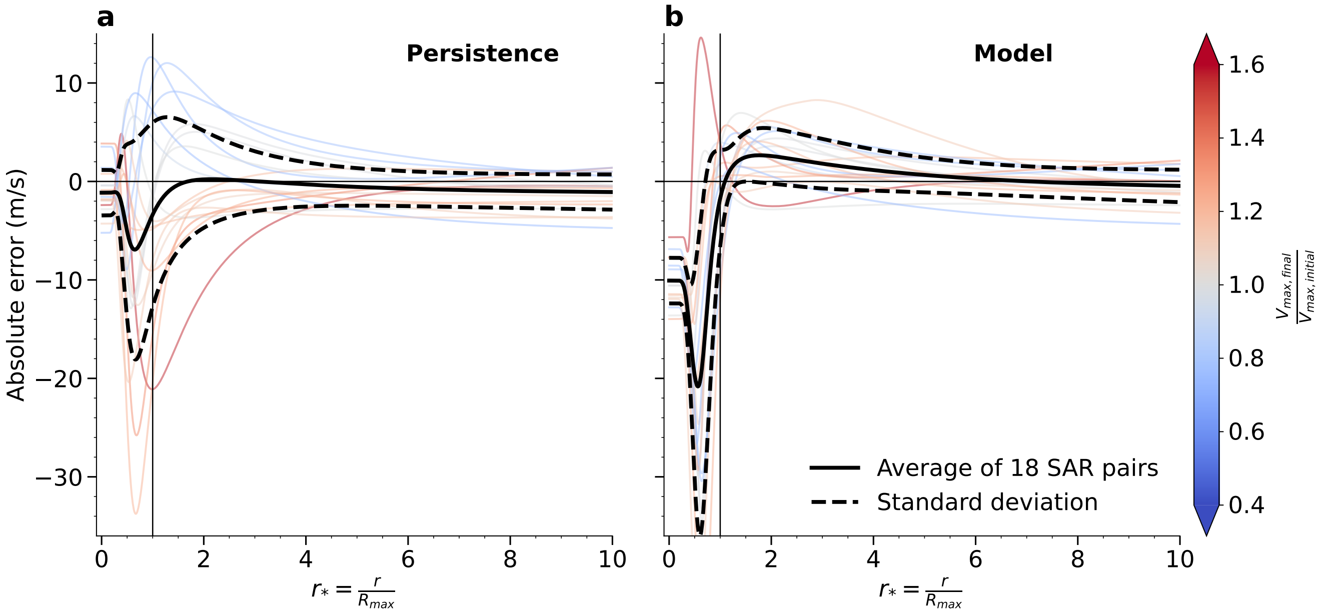

Figure 3. Same as Fig. 2 from the main text, but for absolute error (m/s).

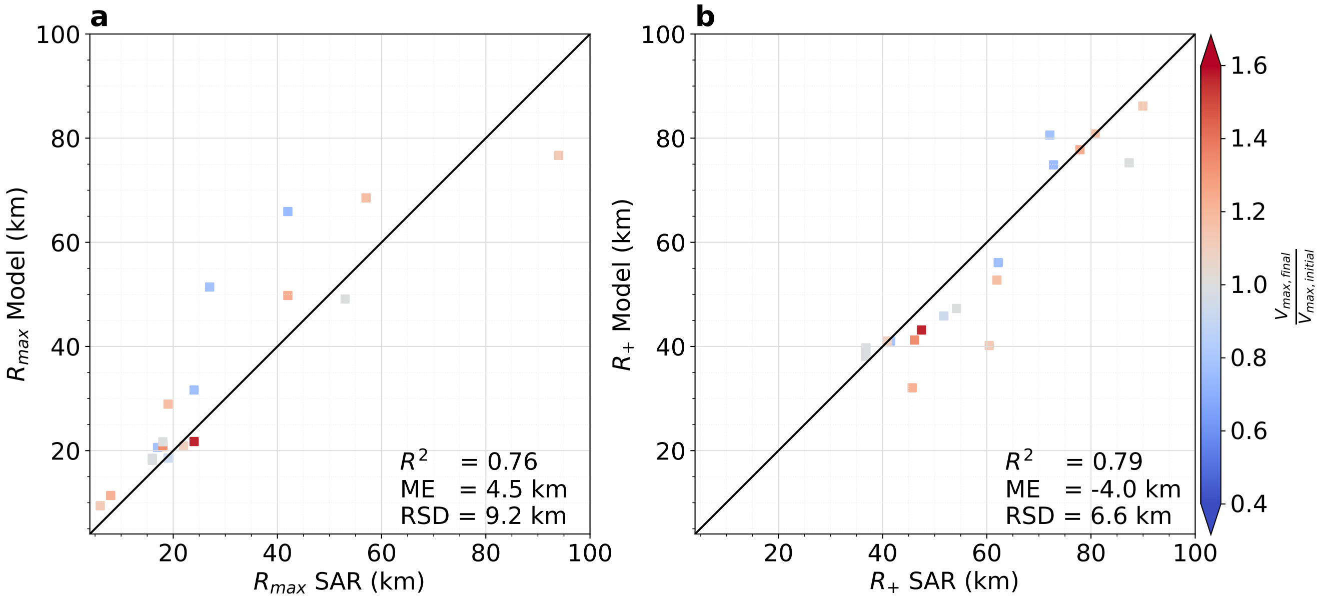

Figure 4. Comparison between model (y-axis) and SAR (x-axis) R_{max} estimates (a), and R_+ estimates (b). The different SAR pairs (squares) are colored by ratio of the final over the initial SAR V_{max} estimates. R^2 is the coefficient of determination, ME the mean error, RSD the residual standard deviation.
